# Follistatin-like Protein 1 Contributes to Acute Traumatic Spinal Cord Injury by Enhancing Neuroinflammation Through the Modulation of Interferon Regulatory Factor 5-Mediated Macrophage Activation

**DOI:** 10.3390/cimb48101065

**Published:** 2026-10-15

**Authors:** Yixing Luo, Shaozhou Ni, Lu Peng, Lili Zhu, Tao Chen, Pengcheng Wang, Zhiqiang Li

**Affiliations:** 1 Emergency Center, Hubei Clinical Research Center for Emergency and Resuscitation, Zhongnan Hospital of Wuhan University, Wuhan 430071, China; 2 Department of Traditional Chinese Medicine (Integrated Traditional Chinese and Western Medicine), Zhongnan Hospital of Wuhan University, Wuhan 430071, China; 3 Department of Biomedical Engineering, Wuhan University TaiKang Medical School, Wuhan 430071, China; 4 Department of Neurosurgery, Zhongnan Hospital of Wuhan University, Wuhan 430071, China

**Keywords:** follistatin-like protein 1, spinal cord injury, macrophage activation, inflammation, interferon regulatory factor 5

## Abstract

**Background and Purpose**: Follistatin-like protein 1 (Fstl1) is a secreted glycoprotein with context-dependent inflammatory activity. We investigated whether Fstl1 is a reproducible feature of traumatic spinal cord injury (SCI) and whether it regulates macrophage activation through interferon regulatory factor 5 (IRF5). **Methods**: A target-blind analysis integrated four public SCI bulk transcriptomic cohorts with single-cell and spatial datasets. Findings were tested in wild-type and Fstl1+/− mice after SCI using spinal cord RNA sequencing, molecular and histological assays, behavioral testing, and bone marrow-derived macrophages with IRF5 gain- and loss-of-function. Adult-induced Postn-CreER-mediated Fstl1 deletion was evaluated in fibroblast–macrophage non-contact co-cultures. **Results**: The public-data screen reduced 440 cross-cohort candidates to four non-structural extracellular genes, including Fstl1. Fstl1 was consistently upregulated across SCI cohorts, correlated with myeloid signatures, and was predominantly associated with non-myeloid cell sources in single-cell data. Experimental SCI increased spinal Fstl1 and inflammatory cytokines, whereas partial Fstl1 depletion reduced tissue damage and improved locomotor recovery. Fibroblast Fstl1 deletion reduced macrophage iNOS, Il6, and Tnf and increased Arg-1 and Il10 in co-culture. Spinal cord RNA sequencing showed broad remodeling of immune and tissue-related programs after Fstl1 depletion. Computational perturbation and experimental validation identified IRF5 as a downstream mediator. Recombinant Fstl1 increased IRF5, iNOS, and CD80 while reducing Arg-1 and CD163; IRF5 silencing reversed these changes. **Conclusions**: Fstl1 is a reproducible SCI-associated extracellular candidate that supports IRF5-dependent inflammatory macrophage activation. Targeting the Fstl1-IRF5 axis may help limit secondary neuroinflammation after SCI.

## 1. Introduction

Spinal cord injury (SCI) causes major motor, sensory, and autonomic dysfunction and can substantially impair quality of life [1,2]. Its pathophysiology involves ischemia, vasogenic edema, excitotoxicity, mitochondrial dysfunction, oxidative stress, neuroinflammation, neuronal apoptosis, and scar formation [1]. Macrophage responses contribute to both secondary injury and repair, and their effects depend on cellular state and the local environment [3]. Multiple immune and inflammatory cell types contribute to neuroinflammation, although their interactions remain incompletely understood. Since neuroinflammation can exert both detrimental and beneficial effects during SCI [3,4], investigating its role in SCI carries substantial clinical relevance.

Follistatin-like protein 1 (Fstl1) is a secreted glycoprotein with context-dependent effects on inflammatory responses. Fstl1 promotes inflammatory reactions involving MAPK and NF-κB signaling in nucleus pulposus cells and chondrocytes [5,6], enhances NLRP3-associated IL-1β secretion in monocytes and macrophages [7], and promotes arthritis through increased IFN-γ expression [8]. Conversely, protective effects have been reported in myocardial ischemia and experimental ischemic stroke [9,10]. Fstl1 expression has also been described during development of the mouse central nervous system [11], but this observation does not establish its inflammatory function after injury. We therefore investigated the expression and functional contribution of Fstl1 in traumatic SCI.

## 2. Materials and Methods

### 2.1. Public Transcriptomic Discovery and Integrative Analysis

A target-blind discovery workflow was applied to public SCI transcriptomic datasets. GSE47681 and GSE45006 served as discovery cohorts. Genes were first required to show persistent upregulation with a key-contrast log2 fold change > 0.9 and were then required to correlate with an independently calculated myeloid score in both cohorts (Spearman ρ > 0.60 and Benjamini–Hochberg FDR < 0.05). Directional replication was assessed in GSE45376 and GSE225308. Retained genes showed positive effects in all four bulk datasets, statistical support in at least three datasets, and a positive random-effects meta-analysis with FDR < 0.05.

Extracellular candidates were defined using GO:0005576, GO:0005615, or GO:0031012. Cell sources were evaluated in GSE162610 by cell-type pseudobulk analysis. Candidates were required to derive at least 75% of compartment-level counts per million from non-myeloid cells. Spatial coupling was tested in nine GSE234774 sections using an inflammatory module that did not contain the candidate gene. Positive correlations were required in at least seven sections, with a positive median ρ and combined FDR < 0.05. Genes annotated as structural extracellular matrix constituents (GO:0005201) were excluded. The final candidates were ranked without gene-specific exemptions using equal weights for bulk effect size, myeloid correlation, non-myeloid source fraction, and spatial inflammatory coupling.

To examine the cellular distribution of Fstl1 beyond the initial discovery analysis, we compared cell-type-resolved expression in four independent mouse SCI single-cell or single-nucleus transcriptomic datasets: GSE162610, GSE172167, GSE196928, and GSE234774. Fstl1 expression was examined separately within each study across the cell populations. The dot plot displays relative mean expression on a within-study scale from 0 to 1. Cell populations without an available estimate are labeled NA. Because sampling regions, injury conditions, and sequencing modalities differed among studies, the analysis was used to compare the cellular distribution of Fstl1 within each study rather than absolute expression between studies.

### 2.2. Animal

Adult male wild-type C57BL/6J mice (Fstl1+/+) were purchased from Vital River Laboratory Animal Technology Co., Ltd. (Vital River, Beijing, China). Fstl1-depleted mice (Fstl1+/−) were generated by Cyagen (Hangzhou, China) as previously described [12]. Mice were backcrossed onto the C57BL/6J background for at least 12 generations before the experiment, and 8-week-old mice were housed under a 12 h light/dark cycle for weight control before the experiment. All procedures were conducted in accordance with the guidelines of Animal Care.

For the cell-source-specific experiments, a Postn-CreER-based conditional Fstl1 knockout line was generated, and recombination was induced in adulthood to target Fstl1 in fibroblasts. Primary fibroblasts isolated from these mice were used in the non-contact co-culture experiments described in the Non-Contact Co-Culture of Primary Fibroblasts and Macrophages section. This adult-induced model complemented the constitutive, whole-body Fstl1 heterozygous model used for the SCI experiments.

### 2.3. Study Design

Mice were randomly assigned to the following 6 groups: (1) a sham-operated group (WT-Sham, *n* = 12): wild-type mice (Fstl1+/+) received a sham operation; (2) a SCI group (WT-SCI, *n* = 12): Fstl1+/+ mice received traumatic spinal cord injury; (3) a Fstl1+/−-Sham group (Fstl1+/−-Sham, *n* = 12): Fstl1-depleted mice (Fstl1+/−) received a sham operation; (4) a Fstl1+/−-SCI group (Fstl1+/−-SCI, *n* = 12): Fstl1+/− mice received traumatic spinal cord injury; (5) a Fstl1+/−-SCI + Vector group (Fstl1+/−-SCI + vector, *n* = 12): Fstl1+/− mice received traumatic spinal cord injury with an intrathecal injection of a lentivirus vector; and (6) a Fstl1+/−-SCI + IRF5 group (Fstl1+/−-SCI + IRF5, *n* = 12): Fstl1+/− mice received traumatic spinal cord injury, and a lentivirus expressing wild-type IRF5 was injected intrathecally into the mice prior to the onset of SCI. The lentivirus expressing wild-type IRF5 (LV11-cmv-neo-IRF5) was prepared by Biofavor Biotech (Wuhan, China) as previously described [13]. The overexpression of IRF5 in the spinal cord was performed by an intrathecal injection of LV11-cmv-neo-IRF5 prior to the onset of SCI. The vector was also intrathecally injected into mice as a negative control. The overexpression efficiency was confirmed by measuring the mRNA and protein expression of IRF5 in injured spinal cords. In each group, 6 animals were killed at 48 h after SCI for biochemical and histological analysis. Another 6 animals in each group were kept for the evaluation of hindlimb motor function and balance ability as described below.

### 2.4. Acute Spinal Cord Injury Model

In the present study, a murine model of SCI was established as previously described [14]. Briefly, mice were anesthetized with 5% sodium pentobarbital (50 mg/kg, i.p.) and placed in the prone position. Then, a laminectomy was performed to expose the T10 spinal cord after skin preparation and positioning. The impact device (6800II; RWD Life Science Corp, Shenzhen, China) was used to create traumatic SCI.

### 2.5. Spinal Cord RNA Sequencing and Enrichment Analysis

At 48 h after SCI, lesion-centered spinal cord tissue from WT-SCI and Fstl1+/−-SCI mice was collected for RNA sequencing (*n* = 4 per group). Total RNA was isolated and assessed for quality before library preparation and sequencing. After removal of adapter and low-quality sequences, reads were aligned to the mouse reference genome and summarized at the gene level. Differential expression was analyzed using DESeq2. Variance-stabilized counts from the 5000 most variable genes were used for principal component analysis. Differentially expressed genes were defined by FDR < 0.05 and an absolute fold change ≥ 1.5 (|log2 fold change| ≥ 0.585).

KEGG and Gene Ontology over-representation analyses were performed separately for genes with positive and negative log2 fold changes. Genes retained after expression filtering were used as the analysis background. Enrichment *p* values were adjusted using the Benjamini–Hochberg method.

### 2.6. Immunofluorescence Analysis of Cultured Macrophages

Bone marrow-derived macrophages were fixed after the indicated treatments, permeabilized, blocked, and incubated with antibodies against CD80 or CD163, followed by fluorophore-conjugated secondary antibodies. Nuclei were counterstained with DAPI. Images were acquired using identical exposure settings across groups. Mean cellular fluorescence intensity was quantified from four independent cultures per group using the same analysis settings for all images.

For the fibroblast–macrophage co-culture experiments, macrophages were stained for iNOS or Arg-1, with DAPI used to visualize nuclei. Fluorescence was quantified as relative target intensity per cell and compared between macrophages co-cultured with control fibroblasts and those co-cultured with fibroblasts carrying adult-induced Postn-CreER-mediated Fstl1 deletion. Four independent biological replicates were analyzed per group.

### 2.7. Basso–Beattie–Bresnahan

The Basso–Beattie–Bresnahan (BBB) motor function score was assessed 1, 3, 7, 14, 21 and 28 days after SCI to evaluate locomotor function recovery [15]. Mice were placed on a circular platform with a diameter of 2 m. The walking and limb activity scores of the hindlimbs were observed and documented. The maximum BBB score is 21 points.

### 2.8. Inclined Plane Test

An inclined plate experiment was performed to evaluate the balance ability of the mice at 1, 3, 7, 14, 21 and 28 days after SCI as previously described [16]. The plane was tilted at a speed of 2°/s, and the angle at which the animal fell from the plane was recorded as the angle of incline.

### 2.9. In Silico Virtual Knockout and Transcriptional Perturbation Analysis

To systematically identify the principal downstream transcriptional targets of Fstl1 in the context of spinal cord injury, an in silico virtual knockout strategy was implemented using publicly available or in-house-generated single-cell RNA sequencing (scRNA-seq) datasets encompassing spinal cord tissue from SCI and sham-operated subjects. Raw count matrices were subjected to standard quality control procedures, including the removal of cells with fewer than 200 detected genes, mitochondrial gene content exceeding 20%, or evidence of multiplet formation based on doublet-detection algorithms. Library size normalization was performed by scaling each cell to a total count of 10,000 reads, followed by log_10_(p) transformation. Virtual knockout simulation of Fstl1 was conducted using the CellOracle framework (v0.12.0) or an equivalent perturbation modeling pipeline, which integrates gene regulatory network inference with transcriptional state propagation to estimate genome-wide expression shifts consequent upon the in silico removal of a defined transcription factor or signaling molecule. Briefly, a cell-type-specific gene regulatory network was reconstructed from chromatin accessibility data (ATAC-seq) or derived from curated prior networks, and signal propagation was modeled iteratively until transcriptional equilibrium was achieved. The perturbation magnitude for each gene was defined as the log_2_ fold-change in predicted expression between the virtual knockout condition and the unperturbed baseline state. Differential perturbation significance was assessed using a permutation-based statistical framework, with *p*-values adjusted for multiple comparisons using the Benjamini–Hochberg false discovery rate (FDR) correction procedure; an FDR-adjusted *p*-value threshold of 0.05 was applied to define statistically significant perturbation responses. Results were visualized using the ggplot2 package (v3.4.0) in R (v4.2.0), encompassing MA-style perturbation landscape plots, volcano plots of perturbation magnitude versus statistical significance, rank-ordered bar charts of top perturbed genes, and integrative scatter plots correlating perturbation magnitude with mean expression level. All bioinformatic analyses were performed using R (v4.2.0) and Python (v3.9). Code and processed data supporting the computational analyses described herein are available from the corresponding author upon reasonable request.

### 2.10. Hematoxylin and Eosin Staining

The T7–T12 spinal cords of mice were dissected 2 days after trauma. All spinal cords were fixed in 4% paraformaldehyde solution and then embedded in paraffin. Then, sections (5 μm in thickness) were taken from the paraffin blocks, and histological examination was performed following a standard HE staining procedure. Then, stained sections were observed under a light microscope.

### 2.11. Nissl Staining

The spinal cord specimens near the lesion epicenter were fixed with formaldehyde, embedded in paraffin and cut into sections (4 μm in thickness). Samples were treated with Nissl staining solution (Boster Biotech, Wuhan, China) for 5 min, and then mounted with neutral balsam. For each section, 5 areas were randomly selected and evaluated under an inverted microscope (Leica, Wetzlar, Germany).

### 2.12. Terminal Transferase-Mediated dUTP Nick End Labeling Assay

Cell apoptosis in the spinal cord was measured by terminal transferase-mediated dUTP nick end labeling (TUNEL) staining in formaldehyde-fixed, paraffin-embedded sections following the manufacturer’s guidelines (Abcam, Shanghai, China).

### 2.13. Immunofluorescence Staining

In order to determine the location of Fstl1 expression in the spinal cord, immunofluorescence staining for Fstl1 in the injured spinal cord was performed as previously described [17].

### 2.14. Macrophage Isolation and Culture

At 48 h after SCI, bone marrow-derived macrophages (BMDMs) were isolated as previously described [18]. The number of cells was counted to ensure that the proportion of positively stained cells used in further experiments was higher than 90%.

### 2.15. Macrophage Stimulation and Assessment of Activation-Associated Markers

Macrophages stimulated with IL-4 plus IL-13 or LPS plus IFN-γ are designated M (IL-4 + IL-13) and M (LPS + IFN-γ), respectively. These terms describe the experimental stimulation conditions rather than mutually exclusive functional identities. Activation-associated gene and protein expression was assessed by RT-qPCR and Western blotting.

#### Non-Contact Co-Culture of Primary Fibroblasts and Macrophages

Primary fibroblasts isolated from control mice or mice with adult-induced Postn-CreER-mediated Fstl1 deletion were co-cultured with macrophages in a two-compartment system. Fibroblasts and macrophages were maintained in separate upper and lower compartments, allowing exchange of soluble factors without direct cell contact. Macrophage stimulation followed the same LPS plus IFN-γ and IL-4 plus IL-13 treatment conditions used in the macrophage-only experiments described above; the corresponding treatments were maintained consistently between the two fibroblast groups. Macrophages were analyzed for iNOS and Arg-1 by immunofluorescence and collected from their compartment for RT-qPCR analysis of Il6, Tnf, Arg1, and Il10. Four independent biological replicates per group were used for immunofluorescence, and three per group were used for RT-qPCR.

### 2.16. IRF5 Gene Silencing in Bone Marrow-Derived Macrophages

Bone marrow-derived macrophages (BMDM) were transfected with IRF5-shRNA (shIRF5, Santa Cruz Biotechnology, Dallas, TX, USA) and negative control shRNA (shCTRL) following the manufacturer’s guidelines. After 48 h of transfection, cells were subjected to the indicated macrophage stimulation conditions with or without exogenous Fstl1 (Abcam, Shanghai, China) at a concentration of 250 ng/mL. The knockdown efficiency was evaluated by measuring IRF5 mRNA and protein expression by RT-PCR and Western blot assays.

### 2.17. Enzyme-Linked Immunosorbent Assay

Inflammatory factors in the injured spinal cord were detected with ELISA kits for TNF-α, IL-1β, and IL-6 (all from Boster Biotech). The SpectraMax M5 Multiplex Microplate Reader (Molecular Devices, Sunnyvale, CA, USA) was used to measure the absorbance values of the ELISA. The measured OD values were converted into concentration values.

### 2.18. Real-Time Quantitative PCR

Total RNA was prepared using a Qiagen RNeasy fibrous mini-kit, using the protocol supplied by the manufacturer, and cDNA was produced using Prime Script RT-PCR Systems (Takara, Otsu, Japan). Quantitative real-time PCR was performed as previously described [12]. The primer sequences used in this study are listed in the Supplementary Materials (Table S1).

For the non-contact co-culture experiment, RNA was extracted from the macrophage compartment, and Il6, Tnf, Arg1, and Il10 expression was measured in three independent biological replicates per group using the RT-qPCR workflow described above.

### 2.19. Western Blot

Western blotting was performed with an SDS-PAGE system as previously described [18]. The primary antibodies used were as follows: anti-Fstl1, anti-iNOS (1:1000), anti-Arg-1, anti-IRF5, and anti-GAPDH (all from Cell Signaling Technology, Danvers, MA, USA). The horseradish peroxidase-conjugated secondary antibody was purchased from Sigma (St. Louis, MO, USA). The enhanced chemiluminescence kit was purchased from Beyotime (Shanghai, China). The relative intensity of protein bands was normalized to GAPDH.

### 2.20. Spectral Flow Cytometry

Spinal cord tissue was collected from wild-type and Fstl1+/− mice at 7 and 14 days after SCI. Fresh tissue was immediately dissociated into single-cell suspensions using dissociation reagents. The freshly prepared suspensions were subjected to antibody staining, followed by spectral flow cytometric acquisition. Antibodies were purchased through Shanghai Universal Biotech Co., Ltd. (Shanghai, China). Surface markers used to identify the populations analyzed here included CD45, CD11b, F4/80, CD3, and CD8.

Intracellular iNOS and ARG1 expression was assessed after permeabilization and intracellular staining. Samples were acquired on a spectral flow cytometer, and data were analyzed using FlowJo v10.

The macrophage-gated population was defined as CD45^+^CD11b^+^F4/80^+^ cells, and CD8^+^ T cells were identified within the CD45^+^CD3^+^ population. Their frequencies were expressed as percentages of CD45^+^ cells. Intracellular iNOS and ARG1 fluorescence intensities were quantified within the macrophage gate.

### 2.21. Co-Immunoprecipitation

Co-immunoprecipitation was performed to examine the association between Fstl1 and IRF5 in macrophages. Following the indicated treatments, cells were lysed in lysis buffer, and clarified lysates were incubated with an anti-IRF5 antibody or a matched control IgG. Immune complexes were recovered using protein A/G beads, washed, and subjected to SDS-PAGE and immunoblotting. The precipitates were probed for Fstl1 to assess its association with IRF5 and for IRF5 to verify immunoprecipitation. Aliquots of the lysates were retained as input controls.

### 2.22. ISRE Luciferase Reporter Assay

An ISRE-driven luciferase reporter assay was performed to assess the effect of Fstl1 on reporter activity in IRF5-overexpressing macrophages. Cells were co-transfected with an IRF5 expression construct and a commercially purchased ISRE luciferase reporter plasmid. All experimental groups overexpressed IRF5. Following transfection, cells were cultured with or without recombinant Fstl1 protein for 24 h before luciferase activity was measured. Reporter activity was expressed relative to the IRF5-overexpressing group without Fstl1 treatment (Control).

### 2.23. Statistical Analysis

The data analysis in the experiment was conducted with SPSS Version 25.0 software (IBM, Armonk, NY, USA). Data were expressed as mean ± standard deviation (SD). Comparisons between two groups were made by unpaired Student’s *t* test. When more than two groups were compared, ANOVA was performed followed by an unpaired Student’s *t* test. A two-tailed *p* value less than 0.05 was considered significant.

## 3. Results

### 3.1. Integrated Public Transcriptomic Analysis Prioritizes Fstl1 as an SCI-Associated Extracellular Candidate

**Figure 1:**
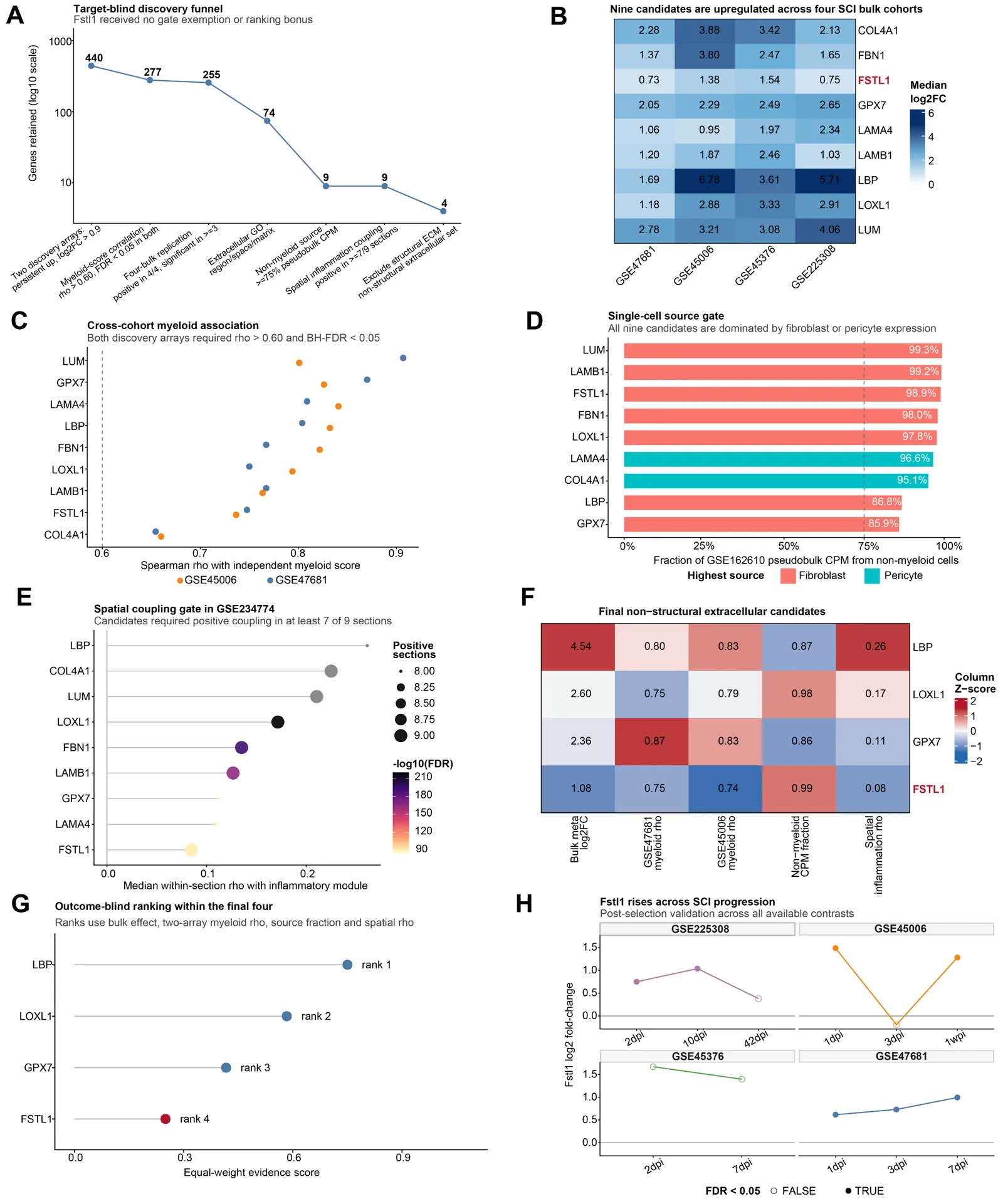
A target-blind, multi-dataset workflow identifies Fstl1 as a reproducible SCI-associated extracellular candidate. (**A**) Sequential screening of genes that were persistently upregulated in two discovery microarray cohorts, correlated with an independent myeloid score in both cohorts, replicated across four bulk datasets, annotated to extracellular compartments, enriched in non-myeloid cell sources, spatially coupled to inflammation, and not classified as structural extracellular matrix constituents. No gene-specific retention rule was applied. (**B**) Median log2 fold changes of the nine candidates retained before the structural matrix exclusion step across GSE47681, GSE45006, GSE45376, and GSE225308. (**C**) Spearman correlations between candidate expression and an independent myeloid score in GSE47681 and GSE45006. (**D**) Fraction of GSE162610 cell-type pseudobulk CPM derived from non-myeloid cells; the dominant source was fibroblasts or pericytes. (**E**) Median within-section correlation with an inflammatory module in nine GSE234774 spatial sections. Point size denotes the number of sections with a positive correlation and color denotes the combined FDR. (**F**) Multi-evidence heatmap of the final four non-structural extracellular candidates; displayed values are unscaled measurements and colors are column Z scores. (**G**) Equal-weight ranking based on bulk effect size, myeloid correlation, non-myeloid source fraction, and spatial inflammatory coupling. (**H**) Fstl1 log2 fold changes across available SCI time points in four bulk datasets. Filled points indicate dataset-specific FDR < 0.05.

The target-blind workflow began with 440 genes that were persistently upregulated in GSE47681 and GSE45006 with a key-contrast log2 fold change > 0.9. Requiring a positive association with an independent myeloid score in both cohorts reduced this set to 277 genes. Replication across four bulk datasets retained 255 genes; extracellular annotation reduced the set to 74, and the single-cell source criterion retained nine. All nine candidates also showed positive spatial coupling to the inflammatory module. Removal of structural extracellular matrix genes yielded LBP, LOXL1, GPX7, and FSTL1 (Figure 1A).

The nine candidates retained before structural-matrix exclusion were upregulated in all four bulk cohorts (Figure 1B). Fstl1 correlated with the myeloid score in GSE47681 (ρ = 0.748, *p* = 5.61 × 10^−4^) and GSE45006 (ρ = 0.737, *p* = 4.07 × 10^−5^) (Figure 1C). In GSE162610, 98.9% of compartment-level Fstl1 expression was assigned to non-myeloid cells, with fibroblasts as the leading source (Figure 1D). Fstl1 was positively coupled to the inflammatory module in all nine spatial sections (median ρ = 0.085; combined FDR = 3.27 × 10^−91^) (Figure 1E).

Multi-evidence integration retained FSTL1 in the final four, although it ranked fourth under the prespecified equal-weight score (Figure 1F,G). Post-selection examination showed increased Fstl1 at multiple acute and subacute time points across the four bulk cohorts (Figure 1H). These independent datasets therefore identified Fstl1 as a reproducible SCI-associated candidate rather than selecting it by prior assumption.

### 3.2. Fstl1 Is Upregulated and Positively Correlates with the Pro-Inflammatory Cytokines Production After SCI

**Figure 2:**
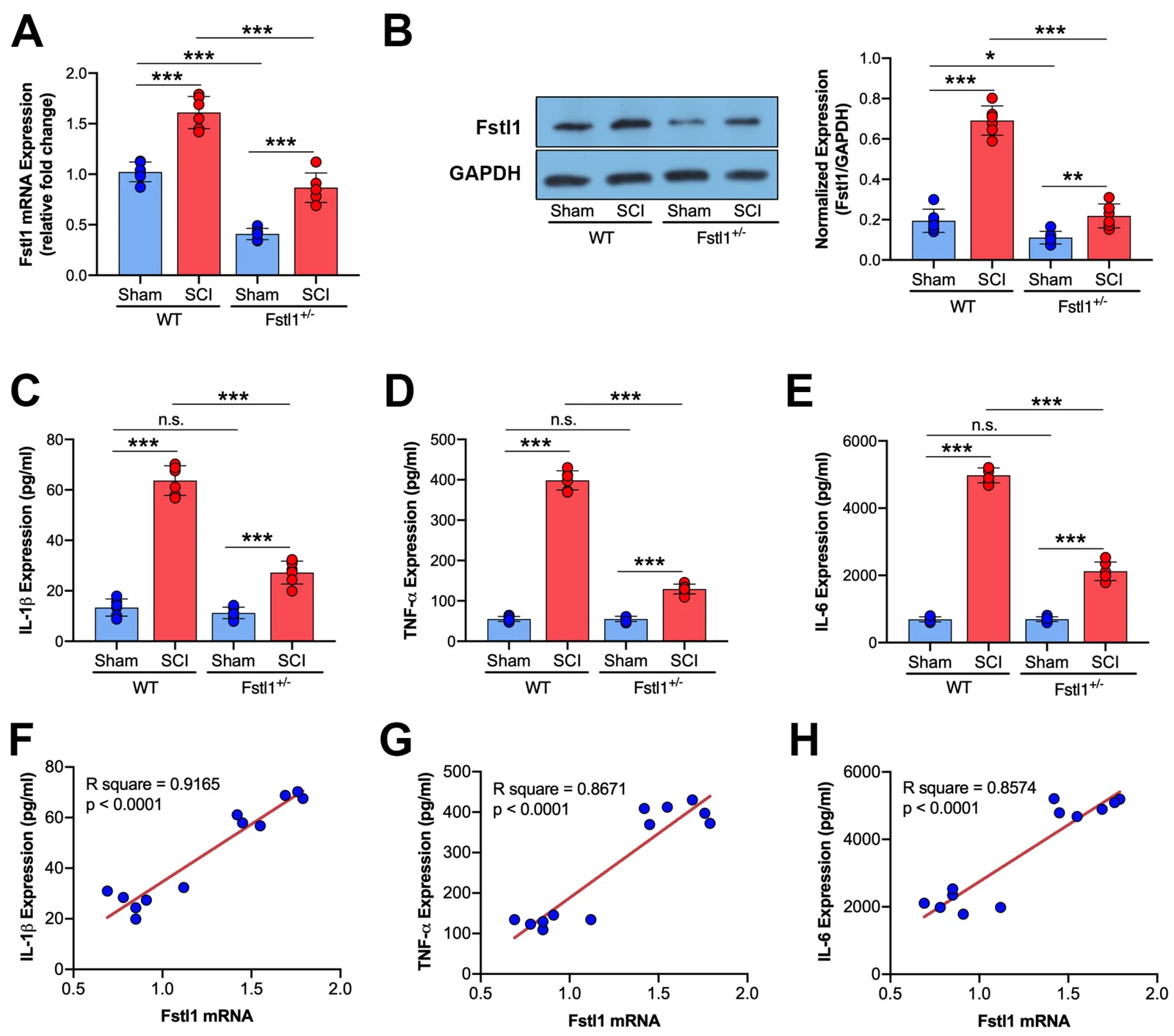
Fstl1 is upregulated and positively correlates with pro-inflammatory cytokine production after SCI. Spinal cord samples were collected at 48 h after SCI for measurement of Fstl1 mRNA (**A**) and protein expression (**B**). ELISA assays were performed for measurement of pro-inflammatory cytokines including IL-1β (**C**), TNF-α (**D**) and IL-6 (**E**) in the spinal cord after SCI. Correlation analysis demonstrated a positive association between Fstl1 mRNA expression and the production of IL-1β (**F**), TNF-α (**G**) and IL-6 (**H**). * *p* < 0.05, ** *p* < 0.01, *** *p* < 0.001 (*n* = 6 per group). n.s. not significant.

We next tested the public-data finding in the mouse SCI model. At 48 h, Fstl1 mRNA and protein levels were higher in WT-SCI spinal cord than in WT-Sham tissue, whereas both measures were reduced in Fstl1+/− mice (Figure 2A,B). SCI also increased IL-1β, TNF-α, and IL-6, and partial Fstl1 depletion lowered each cytokine relative to WT-SCI (Figure 2C–E). Across samples, Fstl1 mRNA was positively associated with IL-1β (R^2^ = 0.9165), TNF-α (R^2^ = 0.8671), and IL-6 (R^2^ = 0.8574), with *p* < 0.001 for all three relationships (Figure 2F–H). These data independently confirmed that increased spinal Fstl1 accompanies the acute inflammatory response to SCI.

### 3.3. Fstl1 Depletion Alleviates SCI and Promotes Locomotor Functional Recovery

**Figure 3:**
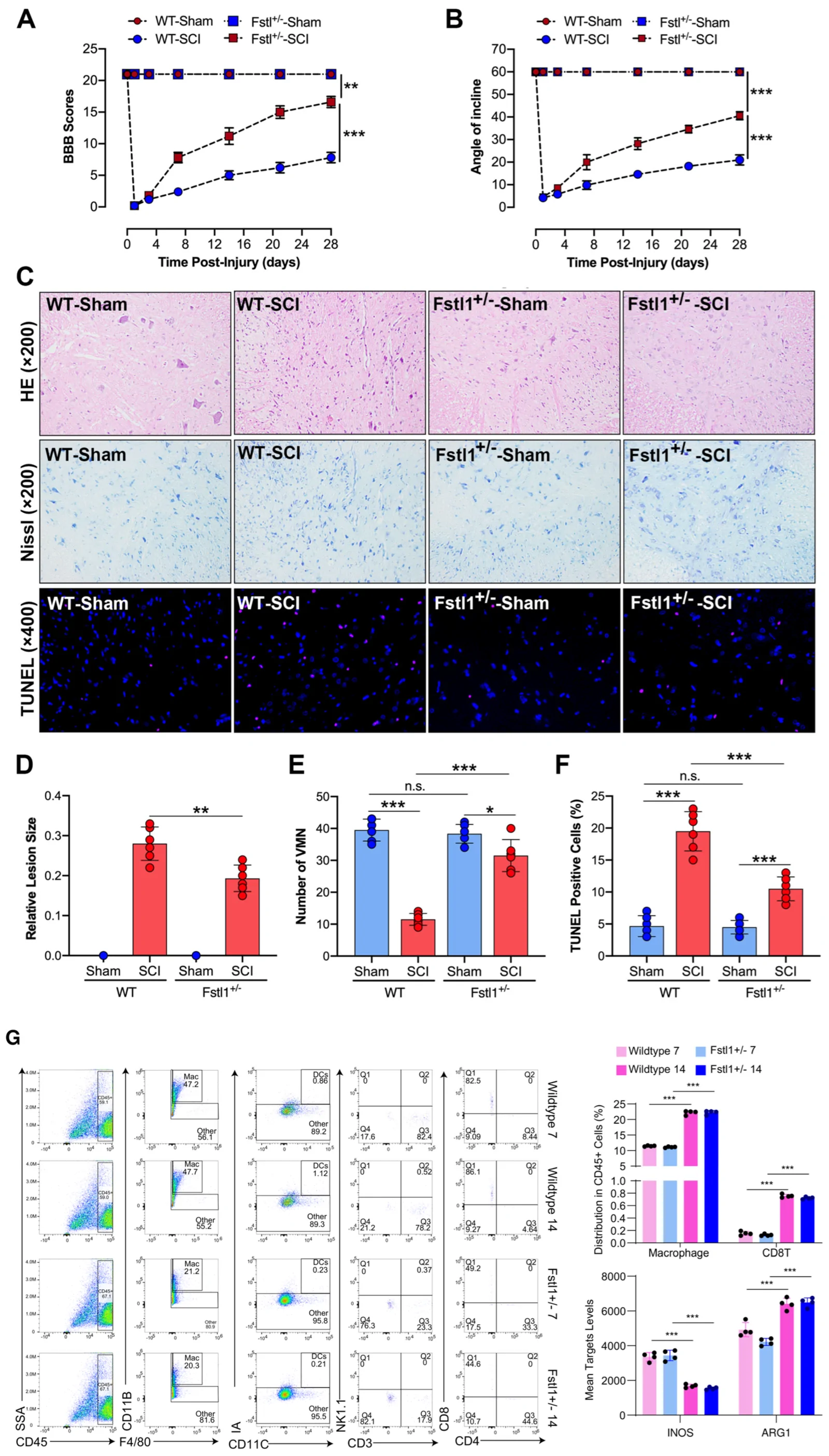
Fstl1 depletion alleviated SCI. The BBB scores (**A**) and the inclined plane test (**B**) were performed to evaluate locomotor function recovery. (**C**) The morphological changes and cell apoptosis in the injured spinal cord were evaluated by HE, Nissl and TUNEL staining. The relative lesion size (**D**), the number of ventral motor neurons (VMNs) (**E**) and the percentage of TUNEL-positive cells (**F**) were measured in WT and Fstl1-depleted mice after SCI. * *p* < 0.05, ** *p* < 0.01, *** *p* < 0.001 (*n* = 6 per group). n.s. not significant. (**G**) Spectral flow cytometric analysis of freshly dissociated spinal cord tissue from wild-type and Fstl1+/− mice at 7 and 14 days after SCI. Left, representative flow cytometry plots showing the gates used to identify CD45^+^ cells, the CD11b^+^ F4/80^+^ macrophage-gated population, and CD3^+^ CD8^+^ T cells. Upper right, percentages of macrophage-gated cells and CD8^+^ T cells among CD45^+^ cells. Lower right, quantification of intracellular iNOS and ARG1 fluorescence intensities within the macrophage gate. Each point represents one independent mouse; *n* = 4 mice per genotype per time point. Data are presented as mean ± SD. *** *p* < 0.001 for the indicated comparisons between days 7 and 14 within the same genotype.

Both WT-SCI and Fstl1+/−-SCI mice developed marked locomotor impairment, but Fstl1+/−-SCI mice achieved higher BBB scores and inclined-plane angles during recovery (Figure 3A,B). Histological analysis showed extensive cavitation, neuronal loss, and apoptosis in WT-SCI spinal cord, whereas these changes were attenuated after partial Fstl1 depletion (Figure 3C). Quantification confirmed a smaller lesion area, greater preservation of ventral motor neurons, and fewer TUNEL-positive cells in Fstl1+/−-SCI mice than in WT-SCI mice (Figure 3D–F). Thus, reduced Fstl1 expression was associated with both structural protection and improved motor outcome.

To extend the assessment of the post-injury immune response, we performed spectral flow cytometry on freshly dissociated spinal cord tissue from wild-type and Fstl1+/− mice at 7 and 14 days after SCI. In both genotypes, the proportions of macrophage-gated cells and CD8^+^ T cells among CD45^+^ cells increased significantly from day 7 to day 14. Within the macrophage gate, intracellular iNOS expression decreased, whereas ARG1 expression increased over the same interval (Figure 3G; *p* < 0.001 for the indicated within-genotype comparisons). These results document time-dependent changes in the composition of recovered immune cells and macrophage-associated marker expression during the subacute phase of SCI.

### 3.4. Adult-Induced Fibroblast Fstl1 Deletion Attenuates Inflammatory Macrophage Activation in Non-Contact Co-Culture

**Figure 4:**
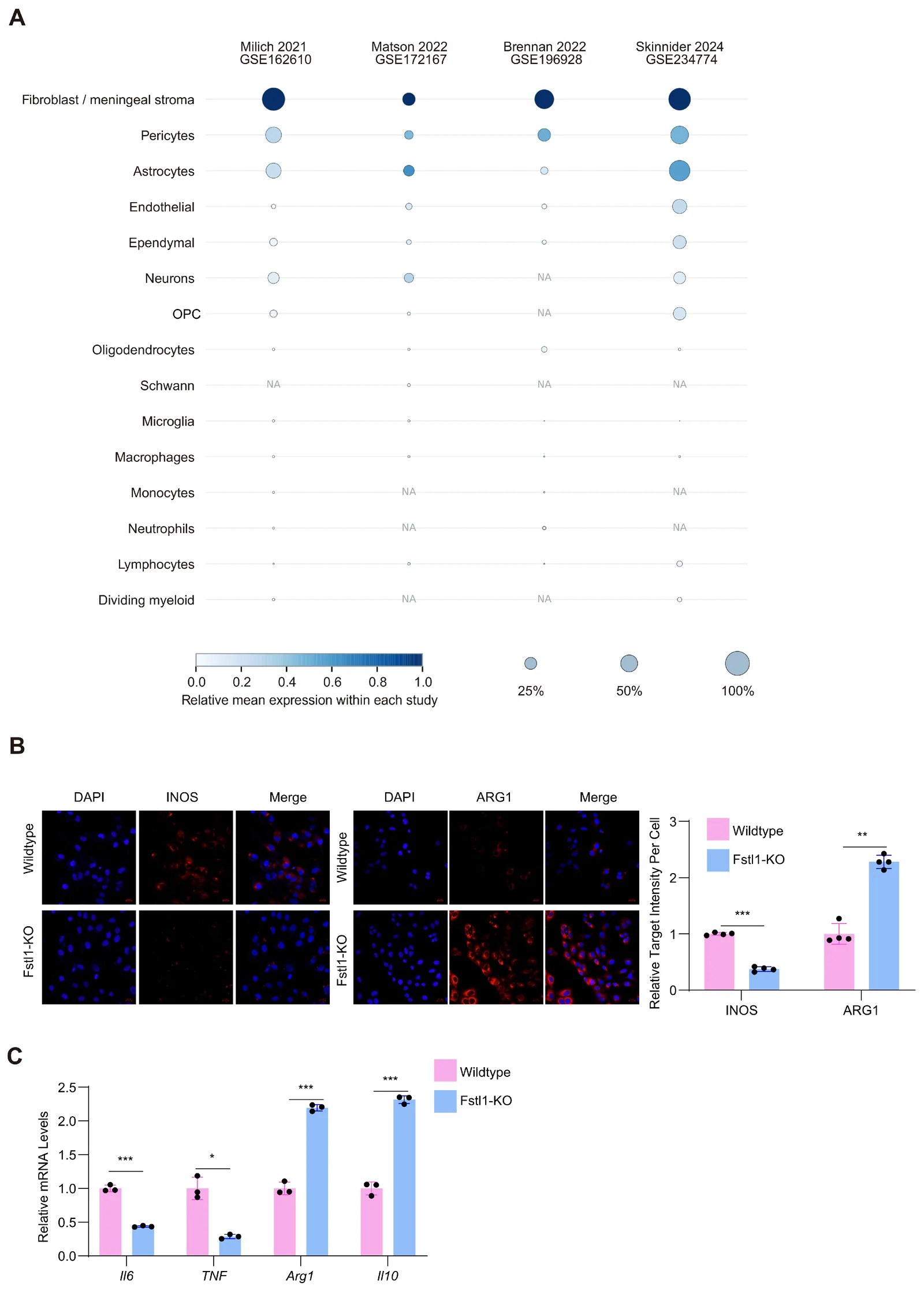
Adult-induced fibroblast Fstl1 deletion alters macrophage activation in non-contact co-culture. (**A**) Cell-type-resolved Fstl1 expression in four mouse SCI datasets: Milich 2021 (GSE162610), Matson 2022 (GSE172167), Brennan 2022 (GSE196928), and Skinnider 2024 (GSE234774). Color denotes relative mean expression within each study (0–1); NA indicates that an estimate is unavailable for the corresponding cell population. (**B**) Representative iNOS and Arg-1 immunofluorescence images and quantification of relative fluorescence intensity per macrophage after non-contact co-culture with control fibroblasts (labeled Wildtype) or fibroblasts carrying adult-induced Postn-CreER-mediated Fstl1 deletion (labeled Fstl1-KO). Nuclei were counterstained with DAPI. (**C**) Relative Il6, Tnf, Arg1, and Il10 mRNA levels in macrophages from the corresponding co-cultures. The group labels refer to the fibroblast genotype. Data are mean ± SD; each point represents an independent biological replicate (*n* = 4 per group in B and *n* = 3 per group in C). * *p* < 0.05, ** *p* < 0.01, *** *p* < 0.001 versus the corresponding control co-culture.

To clarify the cellular source of Fstl1, we examined four independent mouse SCI transcriptomic datasets. The fibroblast/meningeal stromal compartment showed the highest relative mean Fstl1 expression in each dataset, whereas expression was low in microglia, macrophages, and most other immune cell populations (Figure 4A). Expression was also detected in other non-myeloid populations, including pericytes and astrocytes, indicating enrichment in fibroblast/meningeal stromal cells rather than exclusive expression in this compartment.

We therefore generated a Postn-CreER-based conditional Fstl1 knockout line, induced deletion in adulthood, and established non-contact co-cultures using isolated primary fibroblasts and macrophages. Under the corresponding macrophage stimulation conditions, macrophages co-cultured with Fstl1-deficient fibroblasts showed lower iNOS and higher Arg-1 fluorescence intensity than those co-cultured with control fibroblasts (Figure 4B). RT-qPCR showed lower Il6 and Tnf expression and higher Arg1 and Il10 expression in the macrophages co-cultured with Fstl1-deficient fibroblasts (Figure 4C). These findings support a contribution of fibroblast-derived Fstl1 to inflammatory macrophage activation through a non-contact interaction. They establish a cell-source-specific effect in co-culture; the effects of fibroblast-targeted Fstl1 deletion on SCI pathology and functional recovery in vivo require separate evaluation.

### 3.5. Spinal Cord RNA Sequencing Shows Immune and Tissue-Program Remodeling After Fstl1 Depletion

**Figure 5:**
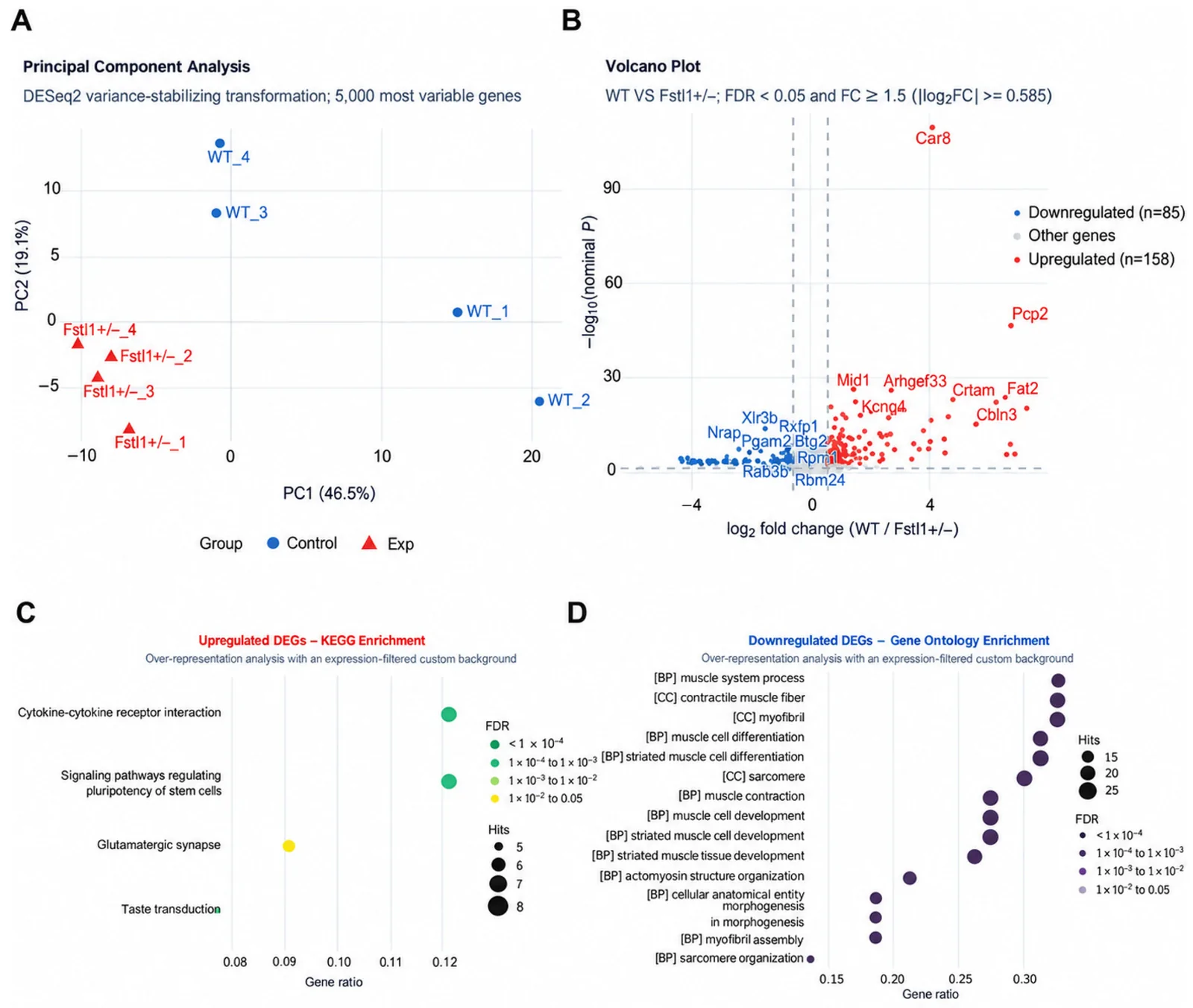
Spinal cord RNA sequencing reveals broad transcriptional changes after partial Fstl1 depletion in SCI. (**A**) Principal component analysis of variance-stabilized counts from the 5000 most variable genes in WT-SCI and Fstl1+/−-SCI spinal cord samples (*n* = 4 per group). (**B**) Volcano plot of differential expression. Genes with FDR < 0.05 and absolute fold change ≥ 1.5 (|log2 fold change| ≥ 0.585) are colored; 158 genes had positive and 85 genes had negative log2 fold changes. (**C**) KEGG over-representation analysis of genes with positive log2 fold changes. (**D**) Gene Ontology over-representation analysis of genes with negative log2 fold changes. Expression-filtered genes were used as the background. Dot position indicates gene ratio, dot size indicates the number of hits, and color indicates FDR.

RNA sequencing was performed on WT-SCI and Fstl1+/−-SCI spinal cords at 48 h. Principal component analysis separated the two genotypes, with PC1 and PC2 explaining 46.5% and 19.1% of the variance, respectively (Figure 5A). At FDR < 0.05 and |log2 fold change| ≥ 0.585, 158 genes had positive and 85 genes had negative log2 fold changes (Figure 5B).

Genes with positive fold changes were enriched for cytokine-cytokine receptor interaction, signaling pathways regulating stem-cell pluripotency, glutamatergic synapse, and taste transduction (Figure 5C). Genes with negative fold changes were enriched for muscle-system processes, contractile fibers, myofibrils, sarcomeres, muscle-cell differentiation, and contraction-related terms (Figure 5D). These results indicate that partial Fstl1 depletion reconfigures both immune signaling and tissue-structural transcriptional programs after SCI.

### 3.6. Computational Perturbation Nominates IRF5 and Experimental Assays Support a Functional Relationship with Fstl1

**Figure 6:**
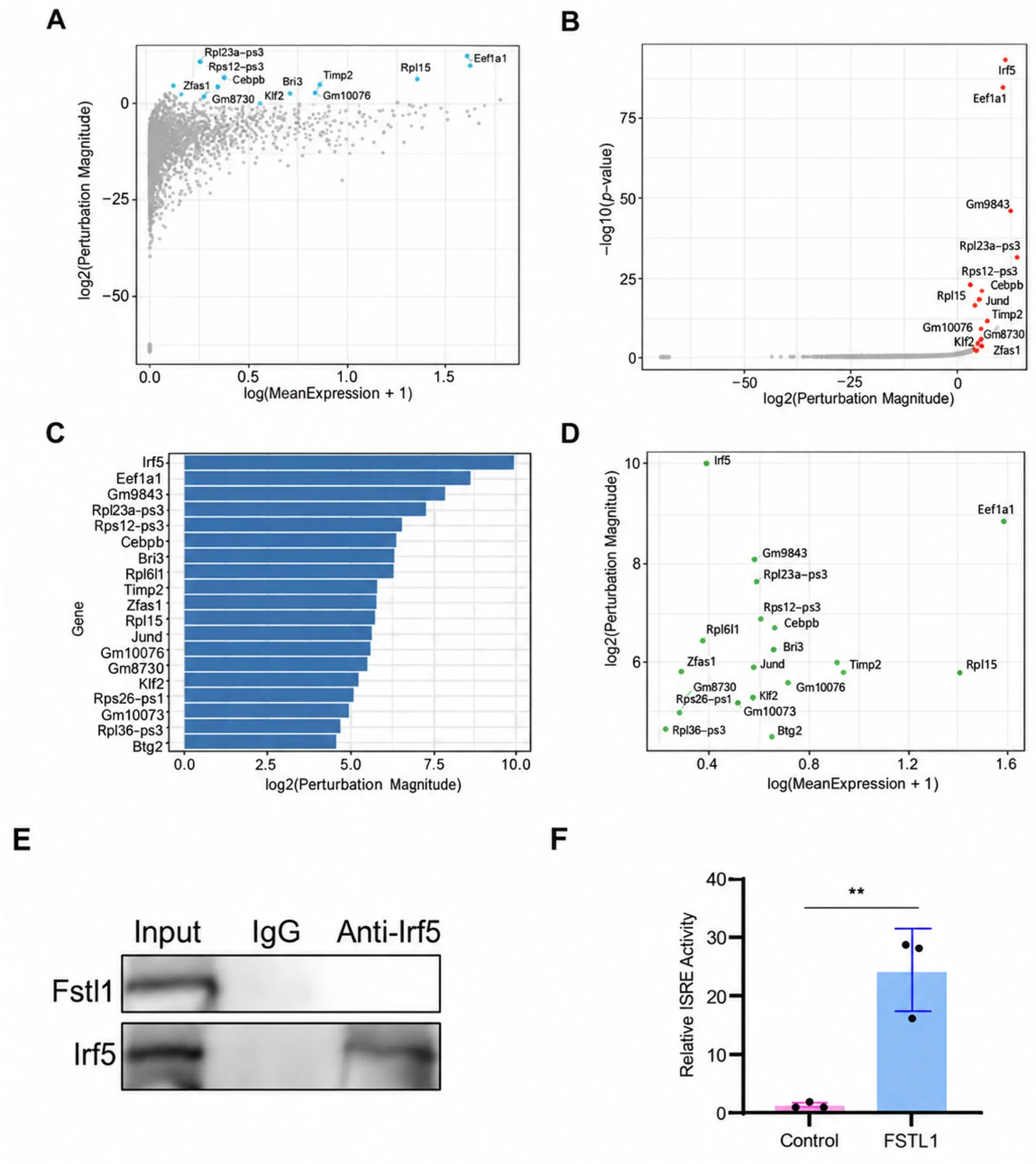
Computational nomination of IRF5 and experimental assessment of its relationship with Fstl1. (**A**) Predicted transcriptional perturbation after virtual Fstl1 knockout plotted against mean expression. (**B**) Perturbation magnitude versus statistical significance; Irf5 is highlighted. (**C**) The 20 genes with the largest predicted perturbations. (**D**) Mean expression and perturbation magnitude for selected candidates. These computational results nominate downstream candidates and do not demonstrate direct regulation. (**E**) Co-immunoprecipitation in macrophages using anti-IRF5 or matched control IgG, followed by immunoblotting for Fstl1 and IRF5. Input lysates were included, and precipitated IRF5 verified recovery. No Fstl1 co-precipitation was detected under the conditions tested. (**F**) ISRE-driven luciferase activity after 24 h with or without Fstl1. Both groups overexpressed IRF5; Control denotes the group without Fstl1. ** *p* < 0.01 for the indicated comparison.

To nominate downstream mediators, we modeled the transcriptional effect of Fstl1 depletion in single-cell data. Most genes showed limited predicted change, whereas a smaller group exhibited larger perturbation magnitudes (Figure 6A). IRF5 combined a large modeled effect with strong statistical support in the genome-wide significance plot (Figure 6B), ranked first among the 20 most perturbed candidates (Figure 6C), and retained a favorable combination of basal expression and perturbation magnitude (Figure 6D). These computational findings nominated IRF5 for direct experimental testing.

Co-immunoprecipitation using an anti-IRF5 antibody did not detect co-precipitated Fstl1, despite recovery of IRF5 in the immunoprecipitates, indicating no detectable Fstl1–IRF5 association under the conditions tested (Figure 6E). To assess the functional relationship, we measured ISRE-driven luciferase activity in IRF5-overexpressing macrophages cultured with or without Fstl1. Fstl1 treatment for 24 h increased reporter activity compared with the IRF5-overexpressing group without Fstl1 treatment (Figure 6F). These findings are consistent with an indirect mechanism through which Fstl1 enhances ISRE-dependent transcriptional activity in IRF5-overexpressing macrophages. The reporter experiment alone does not establish IRF5 dependence because all groups overexpressed IRF5.

### 3.7. Partial Fstl1 Depletion Reduces IRF5 and iNOS Expression and Increases Arg-1 After SCI

**Figure 7:**
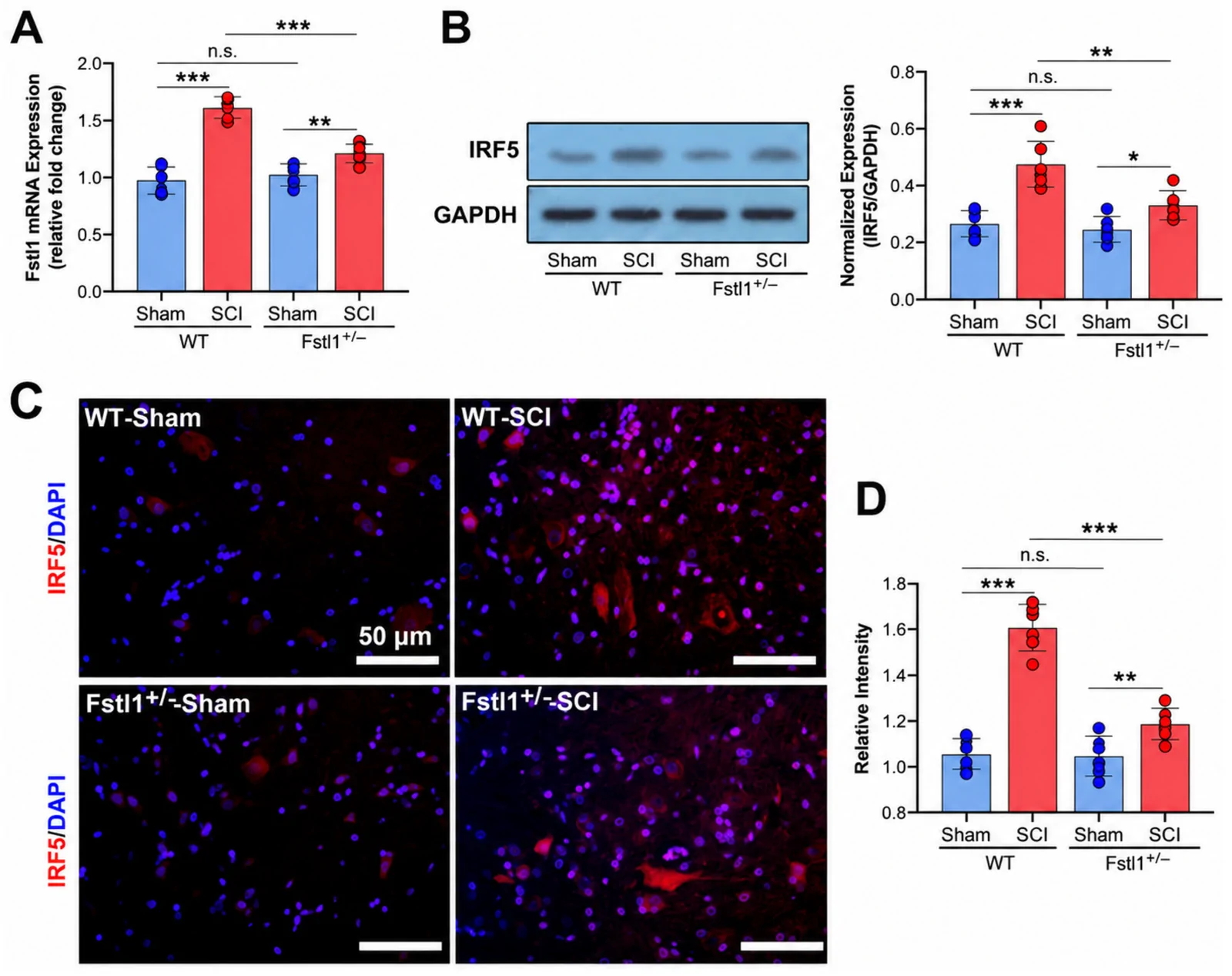
Fstl1 depletion inhibited IRF5 expression in the spinal cord after SCI. RT-PCR (**A**), Western blot (**B**) and immunofluorescence staining (**C**,**D**) were performed to determine IRF5 expression in the spinal cord of WT and Fstl1+/− mice subjected to sham operation and SCI. * *p* < 0.05, ** *p* < 0.01 and *** *p* < 0.001 (*n* = 6 per group). n.s. not significant.

**Figure 8:**
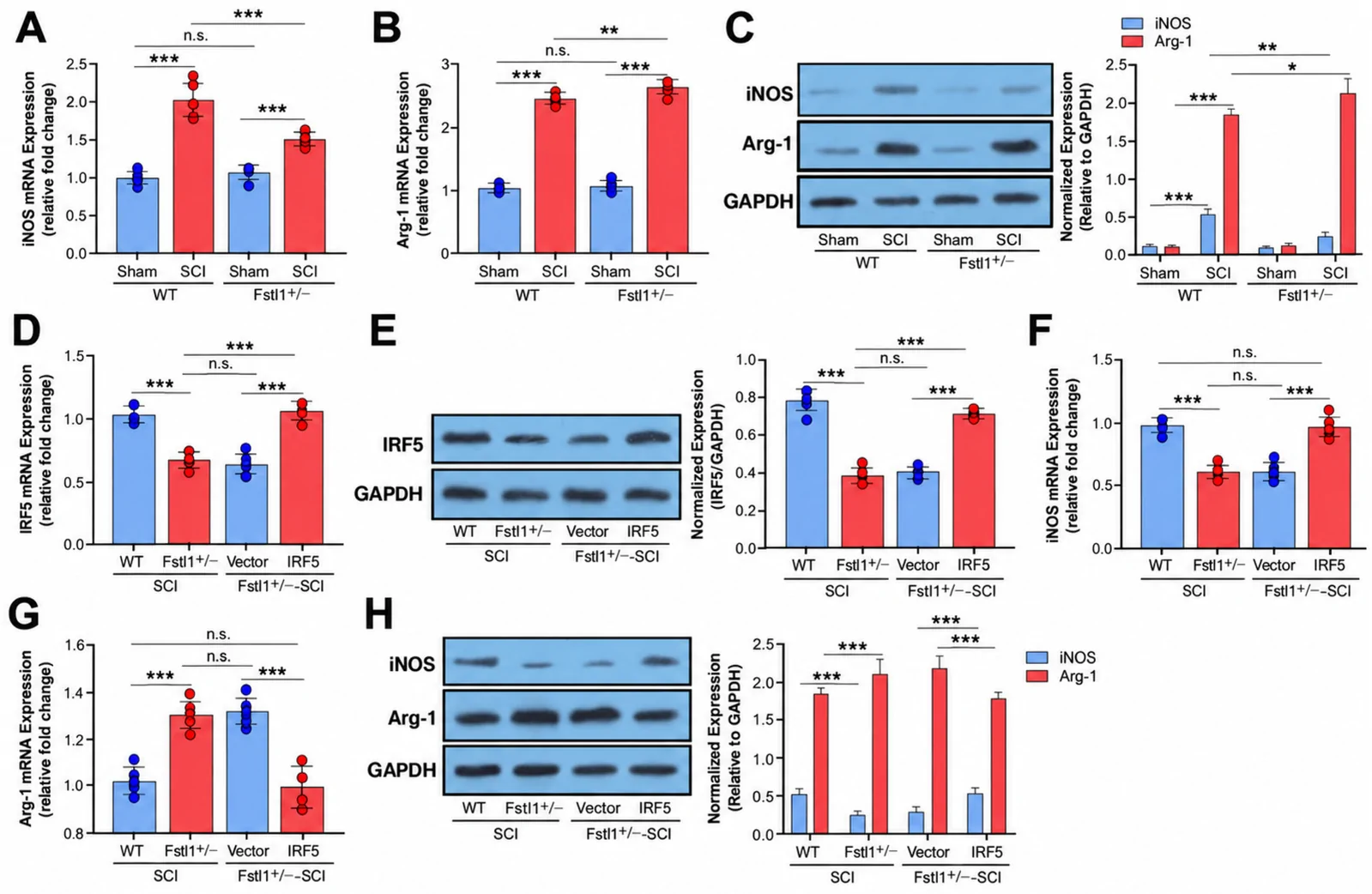
Partial Fstl1 depletion alters iNOS and Arg-1 expression in the injured spinal cord, and these effects are reversed by IRF5 overexpression. The expression levels of iNOS and Arg-1 were measured by RT-PCR and Western blot (**A**–**C**). RT-PCR (**D**) and Western blot (**E**) were performed to measure the expression of IRF5 in SCI mice overexpressing IRF5. (**F**–**H**) The changes in iNOS and Arg-1 mRNA and protein expression were determined by RT-PCR and Western blot after IRF5 overexpression. * *p* < 0.05, ** *p* < 0.01 and *** *p* < 0.001 (*n* = 6 per group). n.s. not significant.

IRF5 mRNA and protein increased after SCI, while both were lower in Fstl1+/−-SCI spinal cord than in WT-SCI tissue (Figure 7A,B). Immunofluorescence and quantitative analysis confirmed the same pattern (Figure 7C,D). In parallel, partial Fstl1 depletion decreased iNOS and increased Arg-1 expression in the spinal cord tissue (Figure 8A–C).

Lentiviral IRF5 delivery restored IRF5 expression in Fstl1+/−-SCI mice (Figure 8D,E) and reversed the effects of Fstl1 depletion on iNOS and Arg-1 (Figure 8F–H). These findings support the involvement of IRF5 in Fstl1-dependent regulation of iNOS and Arg-1 expression in the injured spinal cord.

### 3.8. Exogenous Fstl1 Modulates Macrophage Activation-Associated Markers in an IRF5-Dependent Manner

**Figure 9:**
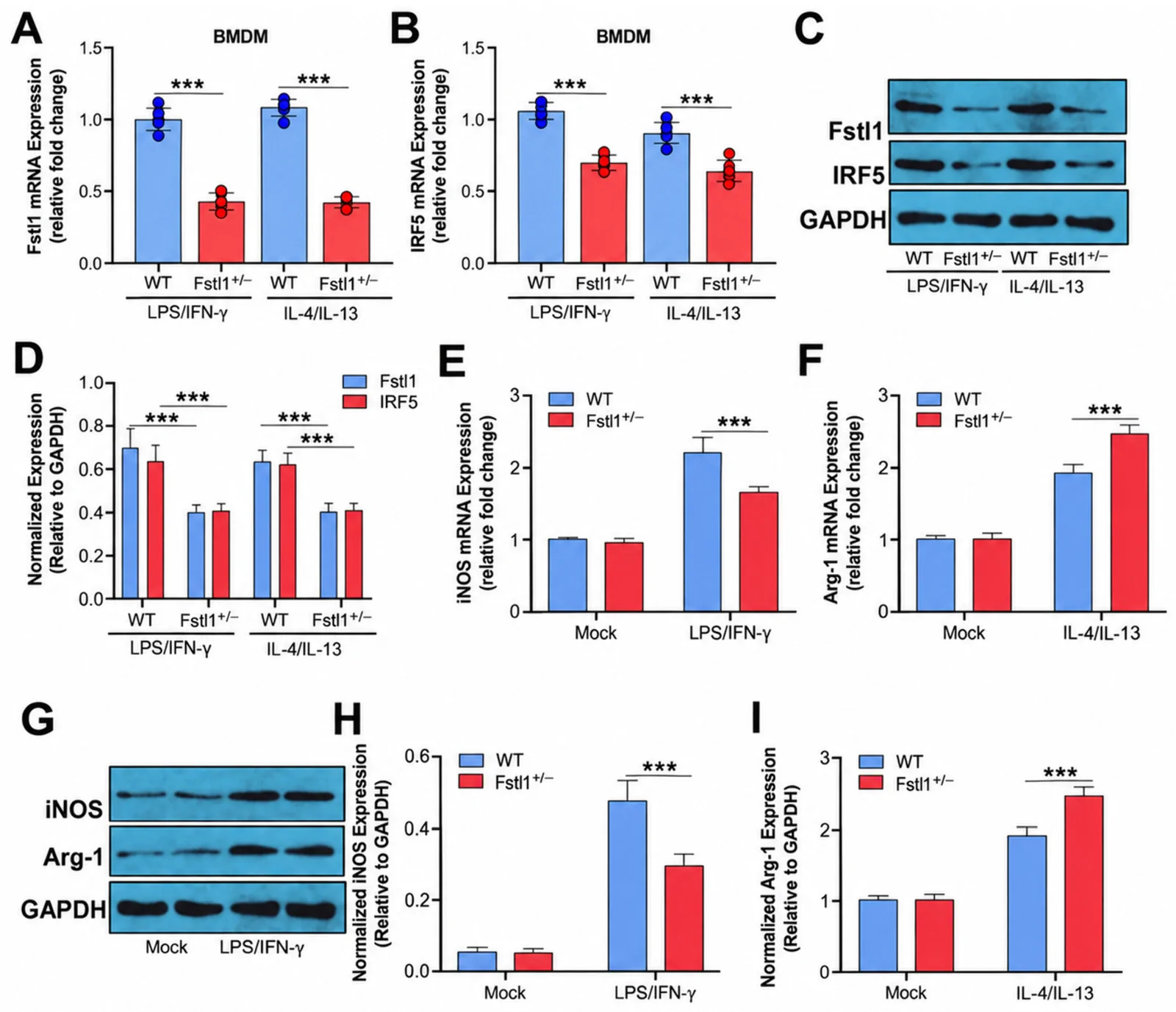
Partial Fstl1 depletion modifies stimulus-dependent iNOS and Arg-1 expression in cultured bone marrow-derived macrophages. RT-PCR (**A**,**B**) and Western blot (**C**,**D**) were performed to determine Fstl1 and IRF5 mRNA and protein expression in BMDM. Then, LPS/IFN-γ stimulation and IL-4/IL-13 stimulation were performed on isolated macrophages. The expression of iNOS and Arg-1 markers was determined by RT-PCR (**E**,**F**) and Western blot (**G**–**I**). *** *p* < 0.001 (*n* = 6 per group).

In bone marrow-derived macrophages, partial Fstl1 depletion reduced Fstl1 and IRF5 expression under the indicated stimulation conditions (Figure 9A–D). Partial Fstl1 depletion reduced iNOS expression following LPS/IFN-γ stimulation and increased Arg-1 expression following IL-4/IL-13 stimulation (Figure 9E–I).

**Figure 10:**
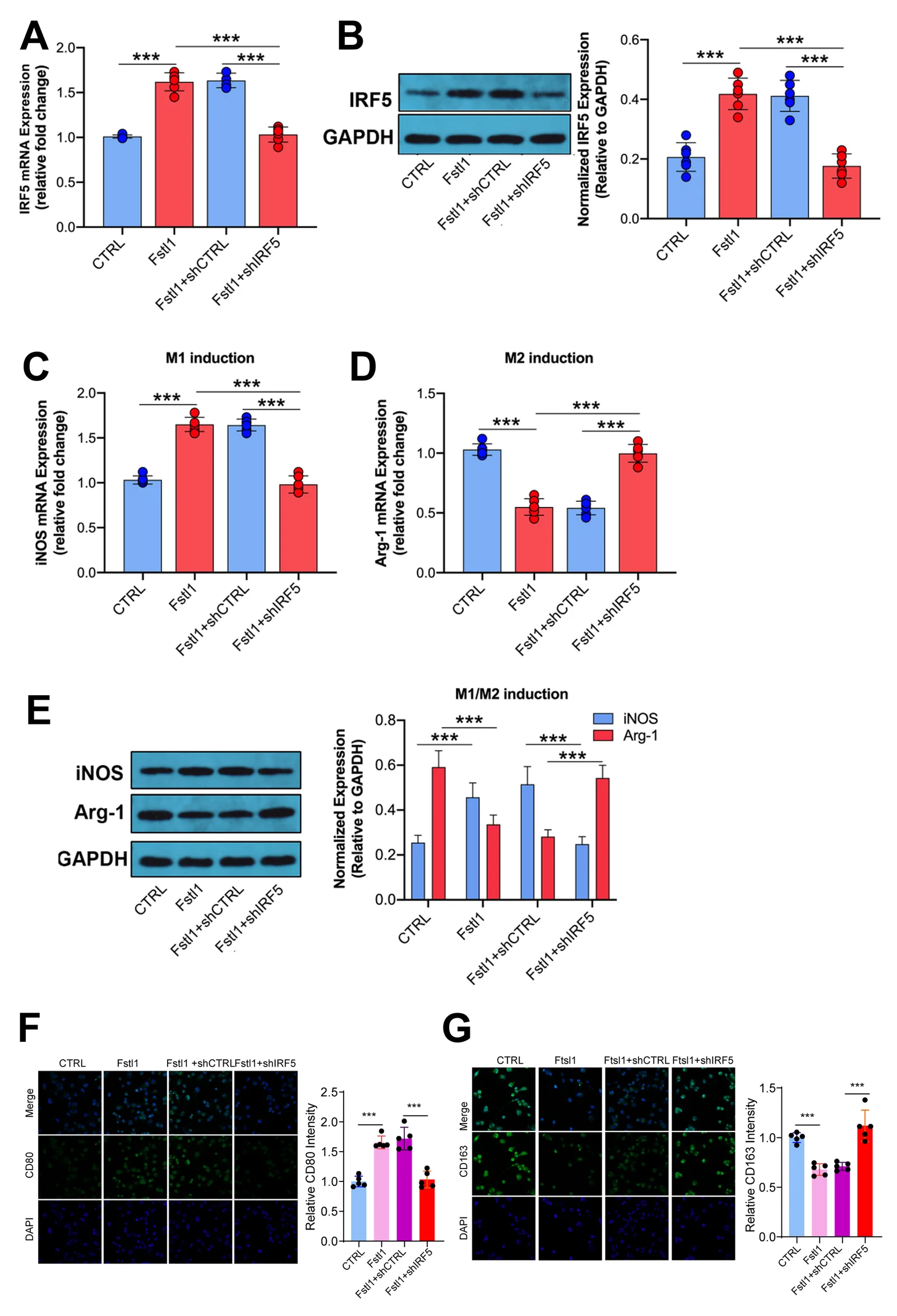
IRF5 silencing reverses Fstl1-induced changes in macrophage activation-associated markers. Isolated macrophages from WT-Sham mice were treated with exogenous Fstl1 under the indicated LPS/IFN-γ or IL-4/IL-13 stimulation conditions. Then, IRF5 mRNA and protein expression were measured by RT-PCR (**A**) and Western blot (**B**). RT-PCR and Western blot were also performed to determine the expression levels of iNOS and Arg-1 (**C**–**E**). (**F**,**G**) Representative CD80 and CD163 immunofluorescence images and quantitative fluorescence intensity; nuclei were counterstained with DAPI. *** *p* < 0.001. For panels (**A**–**E**), *n* = 6 per group; for panels (**F**,**G**), *n* = 4 per group. M1 and M2 labels in the panels denote LPS/IFN-γ and IL-4/IL-13 stimulation, respectively, rather than fixed functional identities.

In the complementary gain-of-function experiment, recombinant Fstl1 increased IRF5 mRNA and protein, whereas shIRF5 prevented this response (Figure 10A,B). Fstl1 increased iNOS and reduced Arg-1, and IRF5 silencing reversed both effects (Figure 10C–E). Immunofluorescence provided an independent phenotypic readout: Fstl1 increased CD80 intensity and decreased CD163 intensity, while shIRF5 returned both markers toward control levels (Figure 10F,G). Together, the loss- and gain-of-function experiments support an IRF5-dependent component of the Fstl1-induced changes in the measured macrophage activation markers.

## 4. Discussion

Our results show that Fstl1 expression increases in spinal cord tissue after SCI and correlates with inflammatory cytokine levels. Previous reports described increased Fstl1 expression and protective activity in myocardial ischemia and experimental ischemic stroke [9,10]. In the present study, partial Fstl1 depletion reduced inflammatory cytokines, tissue damage, and apoptosis and improved locomotor recovery. These findings support a contribution of Fstl1 to inflammatory injury in this experimental setting. However, effects differ across models: recombinant Fstl1 reduces TNF-α-induced inflammatory gene expression in cultured mesangial cells [19], whereas Fstl1 promotes nitric oxide-induced chondrocyte apoptosis through SAPK/JNK/caspase-3 signaling [20]. The SCI findings should therefore be interpreted in their cellular and injury context.

Macrophage responses after SCI comprise heterogeneous activation states with roles in both tissue injury and repair [21]. Although M1/M2 terminology provides a conventional framework, activation states are more precisely described by their inducing stimuli and measured phenotypes [22,23]. Mixed activation features in the injured spinal cord further illustrate the limitations of binary classification [24]. Fstl1-dependent changes in macrophage activation have also been reported in experimental colitis [25]. In our study, partial Fstl1 depletion decreased iNOS and increased Arg-1 expression in the injured spinal cord and altered these markers under defined macrophage stimulation conditions. Recombinant Fstl1 increased iNOS and CD80 and decreased Arg-1 and CD163; IRF5 silencing reversed these changes. These findings support an IRF5-dependent component of the measured macrophage responses. Previous work also linked suppression of IRF5 to altered macrophage activation and improved outcomes in a rat spinal cord ischemia/reperfusion model [13]. Our findings extend this functional relationship to Fstl1 in traumatic SCI. Nevertheless, changes in activation-associated markers do not establish conversion between discrete macrophage populations or demonstrate that cells expressing Arg-1 or CD163 acquire anti-inflammatory or tissue-reparative functions.

Myeloid responses after SCI involve resident microglia, infiltrating neutrophils, and monocyte-derived macrophages, whose functions depend on injury stage and local signals. Early inflammation is not uniformly detrimental: depletion of Ly6G/Gr-1-positive leukocytes impaired wound healing and neurological recovery in mice [4]. Microglia form a protective lesion-border barrier and promote astrocytic scar organization, partly through IGF-1 signaling, thereby limiting inflammatory spread and preserving neural tissue [26]. Macrophage activation states likewise exert divergent effects: persistent inflammatory activation can promote neurotoxicity, whereas repair-associated macrophages support axonal growth in vitro [3]. Recruited monocyte-derived macrophages can also facilitate recovery through IL-10-dependent immunoregulation [27]. Furthermore, comparative single-cell analysis identified four conserved macrophage populations across independent mouse SCI datasets at seven days after injury, highlighting heterogeneity beyond the conventional M1/M2 framework [28]. These findings support interpreting myeloid responses according to cellular origin and functional state rather than classifying them as uniformly harmful or beneficial. In our study, partial Fstl1 depletion reduced inflammatory injury, while IRF5 silencing reversed Fstl1-induced changes in macrophage activation-associated markers, suggesting that Fstl1 promotes an inflammatory activation program. Our source analyses identify fibroblast/meningeal stromal cells as a prominent Fstl1-expressing compartment, with expression also detected in pericytes and astrocytes. Fibroblast-targeted Fstl1 deletion altered macrophage markers in non-contact co-culture, supporting a paracrine contribution from this source. These experiments do not establish the functional contribution of pericyte-derived Fstl1 or whether resident microglia respond similarly. Moreover, global partial Fstl1 depletion and bone marrow-derived macrophage experiments cannot distinguish the contributions of resident microglia and infiltrating macrophages in vivo. Longitudinal, lineage-resolved profiling and cell-specific perturbation are therefore needed to determine whether targeting Fstl1–IRF5 limits detrimental inflammation while preserving phagocytic clearance, lesion containment, and repair.

FSTL1’s opposing effects may reflect differences in receptor engagement, intracellular localization, and the inflammatory state of responding cells. In endothelial cells and cardiomyocytes, DIP2A mediates FSTL1-induced AKT activation and protection against apoptosis [29]. FSTL1 also attenuates myocardial ischemia/reperfusion injury through AMPK activation and inhibition of BMP4–SMAD1/5/8 signaling; AMPK-dependent suppression of inflammatory gene expression has additionally been demonstrated in cultured macrophages [9]. These observations suggest that signaling and metabolic context, rather than tissue identity alone, may determine its net effects. Following SCI, TNF and intracellular iron accumulation sustain inflammatory macrophage activation and hinder reparative polarization [24]. This environment could favor Fstl1-mediated inflammatory amplification, although this interaction remains untested. The fibroblast source analysis and non-contact co-culture results support a paracrine contribution to macrophage activation. Separately, the recombinant Fstl1 and IRF5 perturbation experiments support IRF5-dependent responses in macrophages; whether IRF5 mediates the effects of fibroblast-derived Fstl1 in the co-culture system remains to be tested. Defining their temporal and cell-specific contributions will be necessary to determine whether Fstl1 inhibition can limit detrimental inflammation while preserving protective responses.

Fstl1 may influence macrophage activation through receptor-dependent signaling and metabolic regulation. FSTL1 induces CD14–TLR4-dependent cytokine production, identifying this receptor complex as a candidate mediator of its inflammatory effects [30]. In contrast, FSTL1 can suppress BMP4-induced inflammatory responses through AMPK-associated mechanisms; BMP4 is a signaling ligand rather than an FSTL1 receptor [9]. TLR4 stimulation activates NF-κB and MAPK pathways in macrophages, providing a potential connection between extracellular Fstl1 and inflammatory transcription [31]. Furthermore, studies in TLR7/8-stimulated human monocytes demonstrate that IRAK4–TAK1–IKKβ signaling promotes IRF5 activation, suggesting that signaling components shared with the NF-κB pathway could link Fstl1 to IRF5 without requiring direct protein association [32]. Intracellular FSTL1 also increases mitochondrial ATP production and enhances NLRP3-associated IL-1β secretion, potentially establishing an inflammatory feedback loop that reinforces macrophage activation [7]. We therefore hypothesize that Fstl1 enhances IRF5-associated transcription through receptor-proximal signaling involving NF-κB-related machinery, with NLRP3-dependent cytokine release potentially amplifying this response. This model is compatible with our negative Co-IP result and increased ISRE reporter activity in IRF5-overexpressing macrophages, but neither observation establishes the proposed signaling sequence. Receptor blockade, pathway inhibition, and measurements of IRF5 phosphorylation and nuclear translocation are required to test this mechanism in SCI.

This study has several limitations. First, using only male mice limits generalizability given sex-dependent inflammatory responses after SCI. Second, constitutive, whole-body Fstl1 heterozygous deficiency may permit compensatory adaptation and systemic effects, complicating attribution to acute Fstl1 signaling. Third, most molecular and histological assessments were performed at 48 h. Additional spectral flow cytometry at days 7 and 14 and behavioral follow-up through day 28 extend the temporal assessment but do not provide a complete inflammatory and reparative time course. Flow cytometry quantified macrophage-gated cells and CD8^+^ T cells; neutrophils and microglia were not separately resolved, day-28 flow measurements were unavailable, and spatial relationships were not assessed. Fourth, adult-induced Postn-CreER-mediated Fstl1 deletion now provides fibroblast-targeted evidence in non-contact co-culture, but its effects on lesion pathology and locomotor recovery after SCI have not been established in vivo. This experiment does not exclude compensatory effects in the original whole-body heterozygous model or resolve the relative contributions of resident microglia and infiltrating macrophages. Fifth, the negative Co-IP result does not exclude weak, transient, or condition-dependent association. The ISRE assay, performed entirely in IRF5-overexpressing cells, does not independently establish IRF5 dependence or direct binding to the reporter element. Finally, virtual knockout predictions depend on input data and inferred regulatory networks and should be considered hypothesis-generating. Although IRF5 perturbation supports functional dependence, these predictions do not establish direct Fstl1–IRF5 regulation or identify receptor-mediated mechanisms. Future studies should incorporate both sexes, longitudinal tissue profiling, and in vivo evaluation of adult-induced deletion in defined cellular compartments.

**Figure 11:**
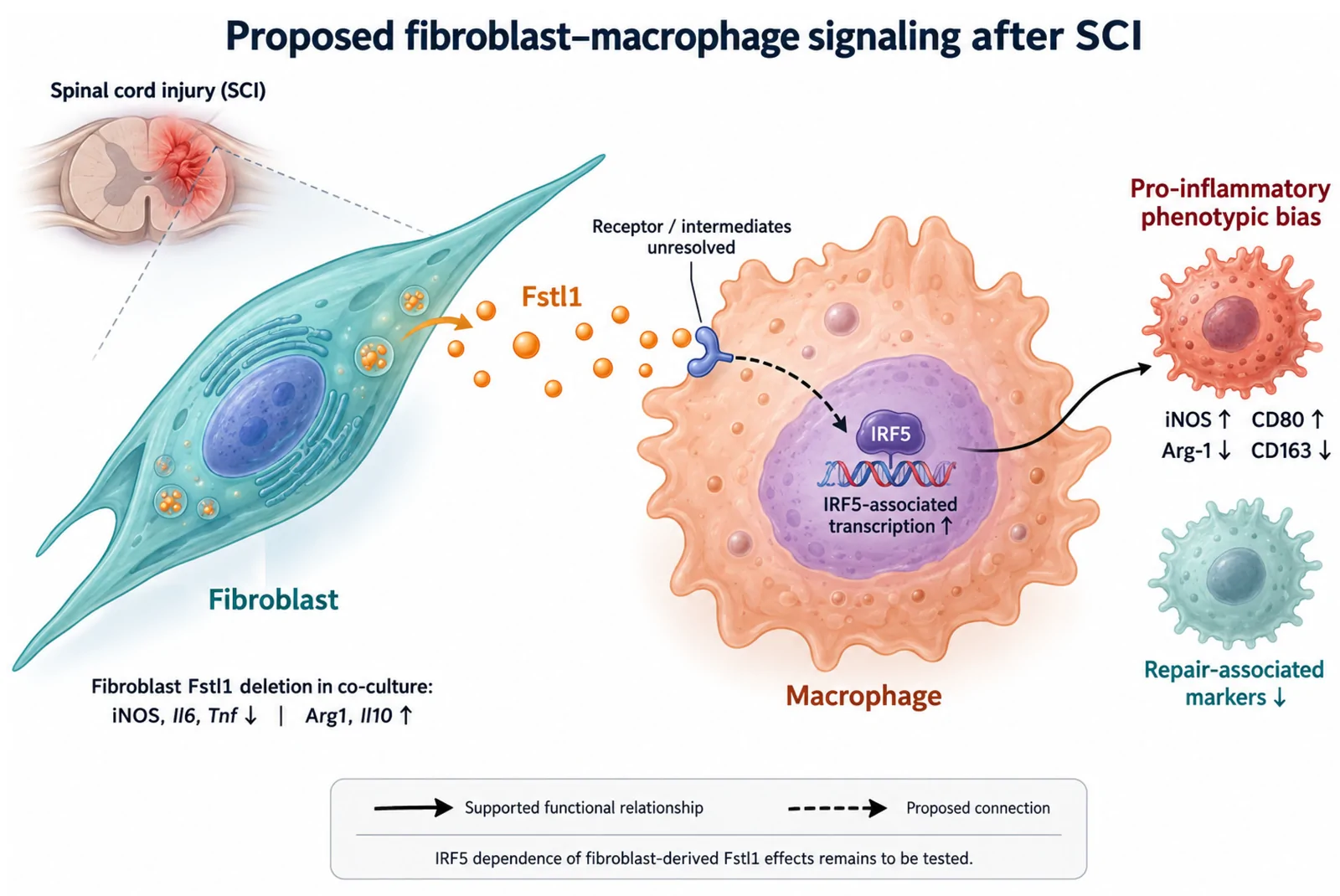
Cellular schematic summarizing Fstl1-associated macrophage activation in the context of spinal cord injury. Cell-type-resolved transcriptomic analyses identified fibroblast/meningeal stromal cells as a prominent Fstl1-expressing population after SCI. In non-contact co-culture, adult-induced Postn-CreER-mediated Fstl1 deletion in fibroblasts reduced macrophage iNOS expression and Il6 and Tnf transcript levels while increasing Arg1 and Il10 expression. In separate macrophage experiments, recombinant Fstl1 increased IRF5 expression, enhanced ISRE reporter activity in IRF5-overexpressing cells, and increased iNOS and CD80 while decreasing Arg-1 and CD163. IRF5 silencing reversed the Fstl1-induced marker changes, supporting an IRF5-dependent component of macrophage activation. The illustrated phenotypic shift represents changes in activation-associated markers rather than conversion between mutually exclusive macrophage states. The schematic integrates separate experimental findings; IRF5 dependence in the fibroblast–macrophage co-culture system was not directly assessed.

The study-supported relationships and candidate upstream mechanisms are summarized in Figure 11.

## 5. Conclusions

Fstl1 expression increased after experimental SCI and was associated with inflammatory cytokine levels. Partial whole-body Fstl1 depletion reduced tissue damage and improved locomotor recovery, while adult-induced fibroblast-targeted deletion altered macrophage activation-associated markers in non-contact co-culture. Separate Fstl1 and IRF5 perturbation experiments support an IRF5-dependent component of the measured macrophage responses. These findings support further investigation of Fstl1 as a potential target for limiting detrimental inflammation after SCI. The upstream receptor, intracellular signaling sequence, and cell-specific contribution to recovery remain to be established.

## Data Availability

All data generated or analyzed during the current study are included in this article. Public datasets analyzed in this study are available from the NCBI Gene Expression Omnibus (https://www.ncbi.nlm.nih.gov/geo/, accessed on 30 August 2026) under accession numbers GSE47681, GSE45006, GSE45376, GSE225308, GSE162610, GSE172167, GSE196928, and GSE234774. Code and processed data supporting the computational analyses are available from the corresponding authors upon reasonable request.

## Supplementary Materials

The following supporting information can be downloaded at: https://www.mdpi.com/article/10.3390/cimb48101065/s1.
