# Supplementary material for: Follistatin-like Protein 1 Contributes to Acute Traumatic Spinal Cord Injury by Enhancing Neuroinflammation Through the Modulation of Interferon Regulatory Factor 5-Mediated Macrophage Activation
Source: Curr Issues Mol Biol. 2026 Oct 15;48(10):1065. doi: 10.3390/cimb48101065 (PMC13703131; doi:10.3390/cimb48101065)
Supplement: Supplementary file 1 [file cimb-48-01065-s001.zip › cimb-4580691-supplementary.pdf]

Table S1 The primer sequences for Real-Time Quantitative PCR

Primer	Sequence (5’-3’)
Fstl1-F	TTATGATGGGCACTGCAAAGAA
Fstl1-R	ACTGCCTTTAGAGAACCAGCC
Il1b-F	GCAACTGTTCCTGAACTCAACT
Il1b-R	ATCTTTTGGGGTCCGTCAACT
Tnf-F	CCCTCACACTCAGATCATCTTCT
Tnf-R	GCTACGACGTGGGCTACAG
Il6-F	TAGTCCTTCCTACCCCAATTTCC
Il6-R	TTGGTCCTTAGCCACTCCTTC
Arg1-F	CTCCAAGCCAAAGTCCTTAGAG
Arg1-R	AGGAGCTGTCATTAGGGACATC
Il10-F	GCTCTTACTGACTGGCATGAG
Il10-R	CGCAGCTCTAGGAGCATGTG
Nos2-F	GTTCTCAGCCCAACAATACAAGA
Nos2-R	GTGGACGGGTCGATGTCAC
Irf5-F	AGAGACAGGGAAGTACACTGAAG
Irf5-R	TGGAAGTCACGGCTTTTGTTAAG
